# Body ownership affects visual perception of object size by rescaling the visual representation of external space

**DOI:** 10.3758/s13414-014-0664-9

**Published:** 2014-05-08

**Authors:** Björn van der Hoort, H. Henrik Ehrsson

**Affiliations:** Department of Neuroscience, Karolinska Institutet, Retziusväg 8, 17177 Stockholm, Sweden

**Keywords:** 3D perception, Cue integration, Multisensory processing, Visual perception, Illusion

## Abstract

Size perception is most often explained by a combination of cues derived from the visual system. However, this traditional cue approach neglects the role of the observer’s body beyond mere visual comparison. In a previous study, we used a full-body illusion to show that objects appear larger and farther away when participants experience a small artificial body as their own and that objects appear smaller and closer when they assume ownership of a large artificial body (“Barbie-doll illusion”; van der Hoort, Guterstam, & Ehrsson, *PLoS ONE*, *6*(5), e20195, 2011). The first aim of the present study was to test the hypothesis that this own-body-size effect is distinct from the role of the seen body as a direct familiar-size cue. To this end, we developed a novel setup that allowed for occlusion of the artificial body during the presentation of test objects. Our results demonstrate that the feeling of ownership of an artificial body can alter the perceived sizes of objects without the need for a visible body. Second, we demonstrate that fixation shifts do not contribute to the own-body-size effect. Third, we show that the effect exists in both peri-personal space and distant extra-personal space. Finally, through a meta-analysis, we demonstrate that the own-body-size effect is independent of and adds to the classical visual familiar-size cue effect. Our results suggest that, by changing body size, the entire spatial layout rescales and new objects are now perceived according to this rescaling, without the need to see the body.

## Introduction

When adults see a toy that they have not seen since their childhood, they tend to be surprised by how small it appears, because they remembered it to be much larger. Anecdotes like this suggest that objects are perceived to be larger to smaller observers and smaller to larger observers. In line with this idea, Poincaré (1952) proposed a thought experiment in which the entire world, including your body, becomes a thousand times larger during sleep. Upon awakening, you would not notice this tremendous change because “our body serves us as a system of axes of coordinates” (Poincaré, 1952, p. 100). A recently published experimental study used a body illusion to confirm Poincaré’s notion that changing body size changes the perceived size of objects (“Barbie-doll illusion”; van der Hoort, Guterstam, & Ehrsson, 2011). The present study sought to examine the possible mechanisms of this *own-body-size effect* by isolating the role of the multisensory experience of owning a body from the mere use of classical visual cues. Because size perception and depth perception are closely related (size is the distance between two sides of an object, and depth is the distance between the object and the observer), we will use the term *space perception* to refer to both.

Psychology textbooks typically explain the visual perception of space on the basis of the *visual cue approach* (e.g., Cutting & Vishton, 1995; Goldstein, 1999; Schwartz, 2010). According to this approach, the perceived size of an object depends on a combination of its size on the retina and its perceived distance from the observer. A multitude of visual cues can be used to perceive object distance. Pictorial cues (e.g., familiar size, relative size, texture gradient, and linear perspective), motion-produced cues (depth from motion and motion parallax), and binocular disparity are all visual cues derived from retinal information. In addition, oculomotor cues (accommodation and eye convergence) are considered visual distance cues because the muscles from which they derive are part of the visual system. The visual cue approach depicts visual perception as being much like a video camera, because it only emphasizes visual cues without accounting for the presence of a body.

However, recent psychological research has revealed that visual perception can also be altered by nonvisual cues that are related to the physiological state of the body (Proffitt, 2006; Proffitt & Linkenauger, 2013). For example, wearing a heavy backpack, fatigue, and poor health make hills appear steeper (Bhalla & Proffitt, 1999) and distances appear larger (Proffitt, Stefanucci, Banton, & Epstein, 2003). Another potential nonvisual cue in visual space perception—the size of the observer—is more difficult to test experimentally (in a within-subjects design) because it would require manipulating the body size of individuals in a laboratory setting. Until recently, the methods that came closest to this requirement altered the apparent size of one’s hand either by zooming in and out on video-recorded displays (Marino, Stucchi, Nava, Haggard, & Maravita, 2010; Pavani & Zampini, 2007) or by using magnifying and minifying goggles (Linkenauger, Ramenzoni, & Proffitt, 2010). It has been shown that such visual distortion of the hand affects both the hand shape during object grasping (Marino et al., 2010) and size perception as measured by a size-matching task (Linkenauger et al., 2010). Interestingly, the latter study found that someone else’s hand does not induce this effect, which suggests that one’s own body or, at least, one’s own hand plays a special role in calibrating size perception. However, these results can largely still be explained by the visual cue approach. First, for the effect of apparent hand size to occur, the hand needs to be visible and located close to the target object, which suggests that the hand is used as a familiar-size cue for direct visual comparison with target objects. In addition, one is more familiar with the sight of one’s own hand, which makes it a stronger familiar-size cue than does someone else’s hand. In addition to the body as an important familiar-size cue, we recently showed that the very sensation of owning a certain-sized body (*body ownership*) is crucial for using the body as a reference in perceiving the sizes of objects (van der Hoort et al., 2011). We used a variation of the *full-body illusion* (Petkova & Ehrsson, 2008) to manipulate the feeling of ownership of different-sized artificial bodies (van der Hoort et al., 2011). The full-body illusion is induced by synchronously touching the participant’s body and the artificial body while participants see the artificial body from a first-person perspective through a set of head-mounted displays (HMDs) connected to a pair of cameras facing down toward the artificial body. Under the control condition, the full-body illusion is significantly diminished by asynchronous visuotactile stimulation. Using this method, we found that identical objects at identical distances were perceived as larger and farther away when participants owned a small body (80 cm) and as smaller and closer when they owned a large body (400 cm). This effect was significantly reduced when body ownership was disrupted by the asynchronous control condition. Importantly, the size of the object on the retina, the binocular disparity when focusing on the object, and the demand characteristics were identical for both the illusory ownership condition and the control condition. Thus, the altered visual perception of size and distance is a consequence of changed body size and is enhanced by body ownership. Therefore, this is referred to as the *own-body-size effect*. We found this own-body-size effect using a variety of measures: verbal estimation of object size and distance, indication of the perceived size with two hands (*bimanual size estimation*), and estimation of the perceived distance by walking that distance blindfolded.

The aim of the present study was to further explore the possible mechanisms and characteristics of this effect. In theory, there are two possible, nonmutually exclusive mechanisms that could contribute to the own-body-size effect. First, body ownership could enhance the effect that results from the artificial body serving as a familiar-size cue for direct visual comparison (i.e., a *direct familiar-size cue*). Disruption of body ownership could decrease the body’s relevance in this respect because a body that is not owned could potentially be of any size and is, thus, less informative. In this case, preventing the possibility of visual comparison between target objects and the artificial body would decrease the own-body-size effect.

The second possible mechanism, which is the hypothesis we propose here, is that ownership of a different-sized body causes a rescaling of external space, which, in turn, causes target objects in this space to be rescaled accordingly. This hypothesis predicts that, after the initial rescaling of external space, the altered size perception of target objects does not depend on visual comparison between the body and those objects.

Our first aim was to disentangle the relative contribution of each mechanism in how body ownership affects visual perception. To this end, we first excluded the influence of the body as a direct familiar-size cue by introducing a new experimental setup that allowed us to occlude the artificial body after induction of the full-body illusion (or a period of asynchronous stimulation in the control condition) but before the presentation of target objects and subsequent size estimations of those objects (Experiment 1). Thus, the crucial difference between this and all previous studies (Haggard & Jundi, 2009; Linkenauger et al., 2010; Marino et al., 2010; van der Hoort et al., 2011) is that no body (or body part) is visible to the participant when the test object is presented.

In Experiment 2, the participants were instructed to look at a fixation point during the period of visuotactile stimulation in order to control for fixation differences. In Experiment 1 (and in our previous study), the participants received no instructions about where to look before the target object presentation. Therefore, the participants could have looked at the artificial body more during the visuotactile stimulation when it was done synchronously, because the visuotactile stimulation may have attracted more attention. This may have caused the artificial body to be a stronger *remembered familiar-size cue* in the body ownership condition and, hence, may partially explain a possible own-body-size effect in Experiment 1. The fixation point in Experiment 2 was located just above the artificial body at a fixed distance from the cameras. Thus, the participants never looked at the artificial body directly, which controlled for the amount of time looking directly at the artificial body. Furthermore, because the distance of the fixation point from the cameras is identical across the body size conditions, an additional effect of this manipulation is that changes in eye vergence—that is, *oculomotor information*—upon the target object presentation are now identical for the various body size conditions. Thus, by controlling for fixation, Experiment 2 controls both for how much participants attend to the body and for oculomotor information from eye-verging muscles.

Comparing the results of these two experiments with those of the previous study (van der Hoort et al., 2011) through a meta-analysis allows us to assess the relative contribution of body visibility and fixation in the own-body-size effect and, thus, disentangles the familiar-size cue hypothesis from the rescaling hypothesis.

The second aim of this study was to characterize the own-body-size effect even further by studying its role in visual size perception at various distances. It is possible that the own-body-size effect applies only to the space immediately surrounding the body (i.e., *peri-personal* space) because the brain represents this space in a special manner. For example, the space near the hands is represented in hand-centered reference frames (Brozzoli, Gentile, Petkova, & Ehrsson, 2011; Farnè, Demattè, & Làdavas, 2005; Graziano, Hu, & Gross, 1997; Spence, Pavani, & Driver, 2004). Furthermore, the mere sight of an object can prepotentiate actions, but only when the object is located close to the body (Cardellicchio, Sinigaglia, & Costantini, 2011). Thus, given that the perception of an object’s size in relation to one's own body is especially important when one is planning to interact with that object, it is reasonable to think that the own-body-size effect on object size perception is stronger in peri-personal space. In contrast, if the own body were to serve as a more fundamental ruler for visual space perception, which is the hypothesis we propose here, one would expect its effect to be equal for near space and far space. Such a result would exclude the possibility that this effect is restricted to space near the body or hands—a possibility that cannot be excluded, on the basis of many of the previously published reports (Haggard & Jundi, 2009; Linkenauger et al., 2010; Marino et al., 2010). By presenting target objects either near the body, in the peri-personal space (1.0 m from the cameras), or far from the body, in distant extrapersonal space (6.0 m from the cameras) (Experiment 3), we could directly compare the body size effect in different parts of space and test our hypothesis.

## General method

### Ethics statement

All participants gave their written informed consent prior to participating in the experiment. All experiments were approved by the Regional Ethical Review Board of Stockholm.

### Participants

We recruited a total of 65 naïve, healthy adult participants for the four experiments, with the following numbers for each experiment: Experiment 1a, 16 (8 females, 8 males, 25.4 years ± 1.1 [*μ*_age_ ± *SE*]); Experiment 1b, 15 (7 females, 8 males, 23.7 years ± 0.8); Experiment 2, 16 (9 females, 7 males, 30.8 years ± 2.4); Experiment 3, 18 (10 females, 8 males, 24.4 years ± 1.0). The participants were recruited by advertisements placed on the campus of Stockholm University, and they received a cinema voucher in exchange for their participation.

We chose to recruit naïve participants for each individual experiment to prevent the participants from adjusting their size estimates according to previous experimental experiences (e.g., estimates of distance between the hands after verbal estimates or vice versa). The use of such a serial design with multiple experiments, instead of a single factorial design with a very large number of conditions, promised to give more reliable results.

### Video technology and artificial bodies

**Fig. 1:**
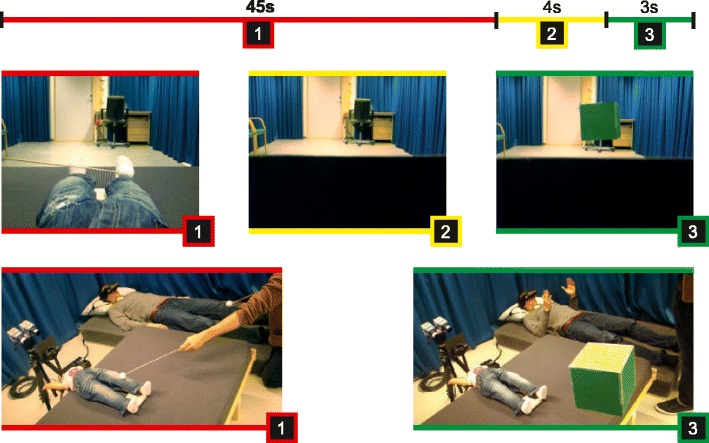
Experimental design. Timeline of a single trial (top), the perspective of the participants through the head-mounted displays (middle), and an overview (bottom) are depicted. There were three periods within each trial: (1) visuotactile stimulation (synchronous or asynchronous), (2) occlusion of the body, and (3) object presentation and estimation. The red fixation ball in the middle panel was visible only in Experiment 2. Only the small-body condition is shown

The participants wore a set of HMDs (CybermindVisette Pro PAL, Cybermind Interactive, Maastricht, the Netherlands; display resolution = 640 × 480, field of view = 71.5°) that were connected to two synchronized color CCTV cameras (Protos IV, Vista, Workingham, Berkshire, U.K.). The distance between the cameras (85 mm) was fixed for all the participants. The image from the cameras was directly transmitted to the HMDs without any software conversion, and so there was no noticeable delay. At the start of the experiment, the participants saw a homogeneous gray screen that was placed a few centimeters in front of the cameras. The first trial began when the experimenter lowered this gray screen, which enabled the participants to see a real-time 3-D image of an artificial body. Two different artificial bodies were used: a small body (80 cm) and a large body (400 cm). Both bodies wore custom-made clothes to match the appearances across the bodies (blue jeans and white socks). The participants could see the legs and lower abdomen of the artificial body and a part of the testing room from a first-person perspective (Fig. 1). Questionnaire data from van der Hoort et al. (2011) showed that the participants noticed the small body to be shorter than the large body, but participants’ average body height estimations (160 and 190 cm, respectively) deviated strongly from the actual height of these artificial bodies.

### Visuotactile stimulation

In this study, we used two visuotactile stimulation conditions. In the synchronous condition, the participants and the artificial body were touched simultaneously at corresponding locations. In the asynchronous condition, we alternately touched the participant’s body and the artificial body on different body parts. The tactile stimulations were random combinations of taps and strokes (of approximately 10–20 cm) applied to the lower legs and feet using a small white ball attached to a rod. The rod was long enough to keep the experimenter’s hand outside the field of view of the cameras. The size of the rod (white ball and stick) and the length of the strokes were proportional to the size of the body being touched in order to optimally match the visual impression of the touches with the tactile sensation (van der Hoort et al., 2011). This procedure ensured that there were no subjectively perceived incongruences between the seen and felt strokes that might otherwise reduce or eliminate the illusion.

### Procedure

Upon arrival, the participants first received instructions in a separate waiting room. In that room, they were then blindfolded and led to the testing room by the experimenter. This procedure prevented them from seeing the true size of the artificial bodies and the target objects in the test room (further information is provided below). The participants lay down on a bed and were fitted with the HMDs. The participants were instructed to keep their eyes closed when the blindfold was removed and the HMDs were fitted; thus, they never saw the room without the HMDs. They were instructed to fold their hands on their chest and keep their head tilted slightly forward (approximately 30°). A gray screen initially occluded the entire field of view of the participant, and the experiment started as soon as this screen in front of the cameras was moved down entirely (by the experimenter). Each trial started with 45 s of synchronous or asynchronous visuotactile stimulation (see Fig. 1). Subsequently, the gray screen in front of the cameras was moved upward again until it covered half of the cameras’ visual fields. The artificial body was now occluded behind the screen, but the upper half of the visual field, which included part of the testing room, remained visible. The target object was then shown at a fixed distance from the cameras (1.2 m) in the half of the visual field that remained visible for 3 s. In total, we used 12 homogeneous colored cubes as target objects, including four 10-cm, four 20-cm, and four 30-cm objects. Various sizes were used to prevent the participants from recognizing object sizes within the conditions and yield a more reliable measure for size perception. Within each group of four, each cube had a different color to prevent the participants from recognizing objects across the different conditions. The time needed to present a target object after occluding the upper half of the cameras’ field of view was approximately 4 s. The participants then indicated the size of the object verbally or with their hands (see below). After this object size estimation procedure, the screen that occluded part of the visual field was lowered again to start a new trial (see Fig. 1).

#### Experiment 1a

The participants were instructed to indicate the size of the cube with their hands (i.e., a *bimanual size estimation*) as soon as the cube was visible. The participants were instructed to keep their hands close to their chest to prevent them from expecting to see their hands in their visual field (of which the lower part was occluded), with the palms turned inward and the fingers fully extended. The distance between the palms was used as a measure of the perceived object size and was manually measured by the experimenter with a ruler. The experiment consisted of four blocks of three trials. Each block contained one condition derived from a full factorial 2 × 2 design: body size (small and large) × visuotactile stimulation (synchronous vs. asynchronous). We used one block for each condition to prevent interference effects across the conditions and to produce the most robust effect possible by maintaining the illusions for a prolonged period of time. Three different object sizes (10, 20, and 30 cm) were used within each block. The order of the three different-sized target objects within each condition and the order of the conditions were randomized across the participants.

#### Experiment 1b

We instructed the participants to verbally report the estimated size of the target objects in centimeters. Each object was presented twice per condition, because we expected greater variability in this measure on the basis of previous findings (van der Hoort et al., 2011); thus, this experiment consisted of 24 trials. The other aspects of the procedure were identical to those in Experiment 1a.

#### Experiment 2

The participants were instructed to look at and maintain fixation on a small red ball (i.e., the fixation point) that was hanging on a fishing line 1.5 m from the cameras. This fixation ball was visible both during the period of visuotactile stimulation and when the artificial body was occluded from view by the screen during the object size estimation procedures. The subsequent presentation of a target object (at 1.2 m from the cameras) occluded the fixation ball entirely, and the participants were instructed to fixate on the target object instead. The conditions and target objects were identical to those in Experiment 1a.

The procedure used in Experiment 2 differs from those in previous studies of the full-body illusion, in which participants looked at the strokes being applied to the artificial body (Petkova & Ehrsson, 2008; Petkova, Khoshnevis, & Ehrsson, 2011; Schmalzl & Ehrsson, 2011; van der Hoort et al., 2011). Thus, to ensure that such a change to the original protocol would not reduce the illusion, we administered a questionnaire to quantify the subjective strength of the illusion. At the end of each condition (i.e., after three trials), the screen in front of the cameras was moved up so that it occluded the entire visual field. The participants were read two statements (test statements T1 and T2) that were designed to capture the subjective experience of the body ownership illusion and two control statements (C3 and C4) that were designed to control for expectancy effects and task compliance (see Table 1; the statements were taken from van der Hoort et al., 2011). The order of the questions was randomized between the conditions and between the participants. The significance of the average differences between the control and test statements was tested for each condition separately.

**Table 1:** Questionnaire included in Experiment 2

Statements		Measure
During this part of the experiment…	T1: … it felt as if the touch I felt was caused by the white ball I saw.	seven point rating scale (“−3” to “+3”)
	T2: … it felt as if the body I saw was my body.	
	C1: … it felt as if I had four legs at the same time.	
	C2: … it felt as if my own body was turning into plastic.	

#### Experiment 3

The participants were informed that objects could appear at various distances and were instructed to estimate the size of each target object with their hands according to the procedures describe above for Experiment 1a. This experiment used another full factorial 2 × 2 design (body size × object distance). We used the same small and large artificial bodies that were employed in the previous experiments, but the visuotactile stimulation was always synchronous in this experiment. Here, the experimental manipulation was the distance at which the target objects were shown from the camera (either 1.0 m in the *near* condition or 6.0 m in the *far* condition) for both body sizes. These distances were chosen because 1.0 m from the camera is within the peri-personal space of both artificial bodies (20 cm beyond the feet of the small body and right above the chest of the large body), and 6.0 m is considered outside the peri-personal space for both bodies. The trials in which the large body was used and the trials in which the small body was used were divided into two separate blocks to prevent inference effects between the small- and large-body conditions, but the six trials within each block were randomized in terms of distance and object size. The order of the two main blocks was randomized across the participants.

### Statistical analysis

For all experiments, we took the weighted average of the responses in each condition. In order to average the size estimations of different object sizes, we multiplied the size estimations of 10 cm objects by 2 and the size estimations of 30 cm objects by 2/3. Collapsing the data from all trials into one condition yields a more precise size estimation per participant and is valid because there is typically no interaction effect of object size on the own-body-size effect (see the “Results” section).

We were not interested in intersubject differences but, instead, in the changed perception within participants; that is, a size estimation increase from 10 to 20 cm in participant A is an identical effect, as compared with a 40–80 cm increase in participant B. Therefore, we standardized the data in such a way that intersubject variability was reduced while intrasubject variability was maintained. For each condition, the difference between a participant's weighted score on that condition and the participant's overall average was divided by this participant’s overall average; *(X*_*i*,A_ *– μ*_A_)*/μ*_A_, where X_*i,*A_ is the weighted score of participant A on condition *i* and *μ*_A_ is the average of participant A on all trials.

These data points were used for further analysis. All the inferential statistics were calculated using SPSS for Windows, release 19.0 (IBM Corp., Armonk, NY). Each data set was first tested for normality with a Kolmogorov–Smirnov test. Experiment 1b and Experiment 3 had at least one condition with significant deviation from normality (*α* = .05) and were, therefore, statistically analyzed using nonparametric tests. We used the Wilcoxon signed-rank test to test for significant differences between individual conditions and to compare these differences (i.e., a nonparametric interaction test). The effect sizes of the nonparametric tests were calculated by *r* ^2^ = *Z*/√*n*.

For the normally distributed data sets of Experiment 1a and Experiment 2, we used repeated measure ANOVAs to test the significance of the main effects and interaction effects. In addition, we performed paired *t*-tests to compare the averages between specific conditions and to compare these differences (this is logically equivalent to examining the interaction term in an ANOVA). The effect sizes of the parametric tests were calculated by *r* ^2^ = *t* ^2^/(*t* ^2^+ *df*).

Finally, we performed a combined analysis of Experiment 1a, Experiment 2, and a previously published experiment (Experiment 6 in van der Hoort et al., 2011) to isolate the effects of body size, body ownership, body visibility, and fixation across different experiments. We used a general linear model in which body ownership and body size were defined as within-subjects factors and body visibility and fixation were defined as between-subjects factors. The *p*-values were corrected (Bonferroni) for multiple comparisons when required.

## Results

### Experiment 1: Body occlusion

**Fig. 2:**
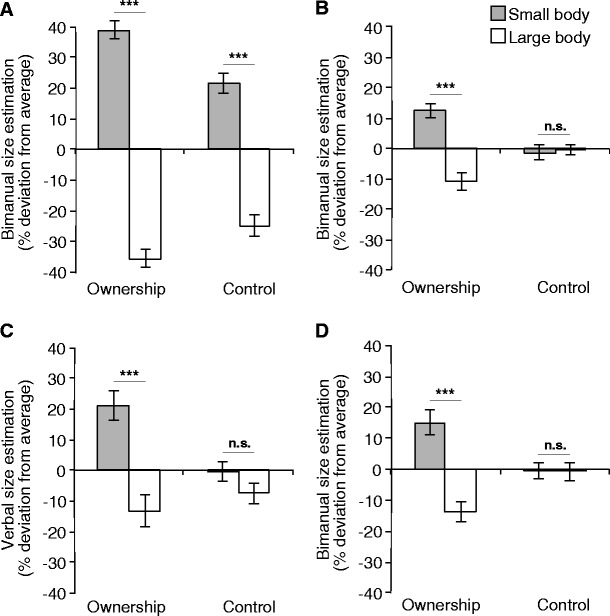
Results of **a** Experiment 6 from van der Hoort, Guterstam, and Ehrsson (2011), **b** Experiment 1a, **c** Experiment 1b, and **d** Experiment 2. Average size estimation per condition is shown as a percentage of deviation from participants’ individual average size estimations. Panels a, b, and d show results from the bimanual size estimation, and panel c displays verbally estimated size. Error bars indicate the *SE*, *** indicates *p* < .001, and n.s. (not significant) indicates *p* > .05

Experiment 1 was designed to test for an own-body-size effect in the absence of a visible body, which excluded the possibility of direct visual comparisons between the object and the body. The crucial finding was that the effect of body size survived this manipulation in the ownership condition. We found a significant interaction effect between body size and body ownership both for bimanual size estimations [Experiment 1a: *F*(1, 15) = 36.185, *p* < .001, *r* ^2^ = .70] and for verbally reported sizes [Experiment 1b: *Z* = 1.988, *n* = 15, *p* < .05, *r* ^2^ = .51], which was based on a significant difference between the small body and the large body in the body ownership condition [Experiment 1a: *t*(15) = 6.408, *p* < .001, *r* ^2^ = .72; Experiment 1b: *Z* = 3.181, *n* = 15, *p* < .001, *r* ^2^ = .82] and the absence of a difference in the control condition [Experiment 1a: *t*(15) = −0.286, *p* = .78; Experiment 1b: *Z* = .966, *n* = 15, *p* = .33] (see Fig. 2b, c). This interaction effect did not depend on the size of the object presented at a given trial for Experiment 1a, F(2, 30) = 0.307, p = .74. However, object size had a close to significant effect on the verbal size estimations in Experiment 1b, χ^2^ = 5.897, *n* = 15, *p* = .052, based on a larger own-body-size effect for the 20-cm objects.

Overall, the results of Experiment 1 show that the participants estimated objects to be larger when they experienced having a small body and estimated objects to be smaller when they experienced having a large body, but not when the sense of body ownership was absent. Furthermore, we found robust effect sizes for both the bimanual estimations (Experiment 1a) and the verbal reports (Experiment 1b). However, because of large interindividual differences in verbal estimations and an associated lack of normality in the acquired data sets (also seen in van der Hoort et al., 2011), we used the bimanual size estimation in subsequent experiments, which tended to yield normally distributed responses, to allow for parametric analyses.

### Experiment 2: Body occlusion and fixation

The second experiment was designed to examine the own-body-size effect in the absence of both direct visual comparison and fixation differences. The rationale of Experiment 2 was to control for the amount participants fixated directly on the artificial body while maintaining the crucial experimental manipulation of occluding the body from view that was used in Experiment 1. To this end, the procedure was identical to that in Experiment 1, with one exception: The participants were instructed to focus on a red fixation ball (at 1.5 m from the cameras in all conditions) during the visuotactile stimulation period. The subsequent presentation of target objects (always at 1.2 m) occurred in front of the fixation ball. The postexperiment reports revealed that 1 participant failed to maintain fixation and was, therefore, excluded from further analyses.

Consistent with the results of Experiment 1a, the size estimations showed a significant interaction effect between body size and synchronicity, *F*(1, 14) = 20.579, *p* < .001, *r* ^2^ = .58, which was caused by a significant difference between body sizes in the body ownership condition, *t*(14) = 5.246, *p* < .001, *r* ^2^ = .65, and an absence of this difference in the control condition, *t*(14) = 0.047, *p* = .96 (see Fig. 2d). This interaction did not depend on object size, *F*(2, 30) = 1.051, *p* = .36.

**Fig. 3:**
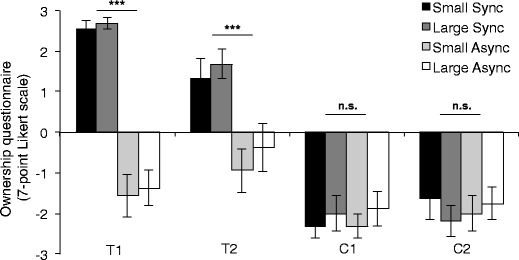
Questionnaire data from Experiment 2 for each of four conditions: body size (small or large) and type of visuotactile stimulation (synchronous or asynchronous). T1 = test statement 1: “It seemed as if the touch I felt was caused by the white ball.” T2 = test statement 2: “It seemed as if the body I saw was my body.” C1 = control statement 1: “It seemed as if my own body was turning into plastic.” C2 = control statement 2: “It seemed as if I had four legs at the same time.” Error bars indicate *SE*

We had the participants complete a very short body ownership questionnaire (van der Hoort et al., 2011) after each of the four conditions of Experiment 2. The rationale for this was to demonstrate that the illusion was fully established even though the participants were not directly looking at the artificial body and the strokes applied to it were as in previously published studies. Importantly, the participants’ responses showed significant differences between the two ownership statements and the two control statements for both the small body and the large body, but only in the ownership condition, *Z*_small_ = −3.418, *n* = 15, *p* < .001; *Z*_large_ = −3.524, *n* = 15, *p* < .001, and not in the control condition, *Z*_small_ = −1.401, *n* = 15, *p* = .16; *Z*_large_ = −1.684, *n* =15, *p* = .09 (see Fig. 3). Furthermore, these differences were significant, *Z*_small_ = −3.371, *n* = 15, *p* < .001; *Z*_large_ = −3.244, *n* = 15, *p* < .001, which indicates a stronger sense of ownership in the ownership condition than in the control condition. These results are in line with previously published results (van der Hoort et al., 2011).

**Fig. 4:**
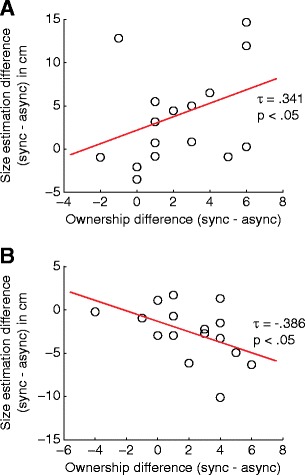
Correlations between body ownership ratings and size estimations for **a** the small-body condition and **b** the large-body condition. The *x*-axis reflects the difference between average size estimations on synchronous (sync) and asynchronous (async) trials. The *y*-axis reflects the difference between the synchronous and asynchronous conditions for participants’ rating of the statement “It felt as if the body I saw was my body” (T2 in questionnaire)

Thus, the effect of visuotactile synchronicity on body-dependent size perception was accompanied by an effect on subjective body ownership (statement T2). To strengthen the evidence for the necessity of body ownership in the own-body-size effect, we performed a post hoc correlation analysis between the change in subjective body ownership (T2_sync_ − T2_async_) and the change in perceived object size (*μ*_sync_ − *μ*_async_). Interestingly, participants’ change in perception correlated with their change in subjective body ownership for both body size conditions (Kendall rank correlations: *τ*_small_ = .341, *N* = 16, *p* < .05; *τ*_large_ = −.386, *N* = 16, *p* < .05) (see Fig. 4). Thus, the stronger a participant experienced the small-body illusion, the bigger target objects appeared, and the stronger a participants experienced the large-body illusion, the smaller target objects appeared.

### Experiment 3: Different parts of space

In the last experiment, we examined the own-body-size effect in different parts of space: the extrapersonal space near the body (peri-personal space; 1 m) and the extrapersonal space far from the body (“far space”; 6 m). On the basis of earlier studies (Cardellicchio et al., 2011; Haggard & Jundi, 2009; Linkenauger et al., 2010; Marino et al., 2010), a hypothesis could be formulated in which one would expect a larger effect for objects presented in peri-personal space, as compared with those presented in far space. This hypothesis contrasts with our hypothesis that the own body is used as a fundamental ruler for visual space perception in general. Our hypothesis predicts equal effects for objects presented in peri-personal and far space.

**Fig. 5:**
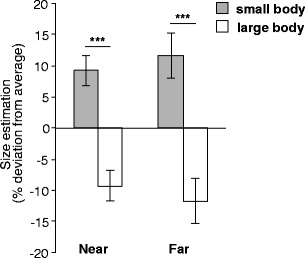
Results of Experiment 3: Average size estimation according to distance and body size as a percentage of deviation from the participants’ individual average size estimations of that distance

**Fig. 6:**
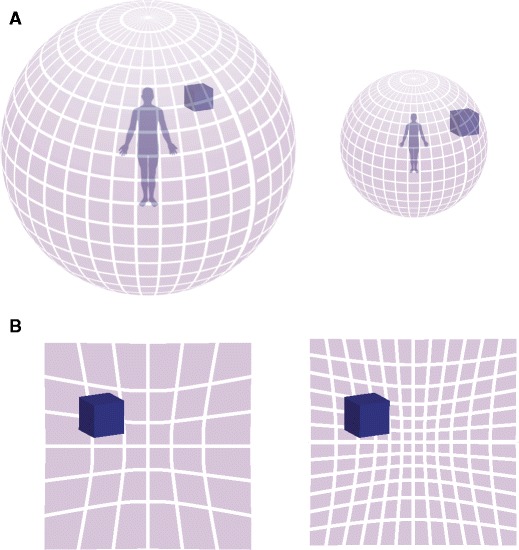
Model of the own-body-size effect. **a** The axes of coordinates of world space are defined by the size of the body, which makes a smaller observer perceive the distance of an object to be larger (more grid-lines between the observer and object) and **b**) the size of an object to be larger (more grid-lines occluded by the object) despite identical retinal input

To test this prediction, we used a full factorial 2 × 2 design in which the target objects were presented at either 1.0-m (near) or 6.0-m (far) distances from the cameras in both body size conditions. The results showed that the own-body-size effect was very similar for both distances (near: *Z* = 2.940, *n* = 18, *p* < .001, *r* ^2^ = .70; far: *Z* = 3.420, *p* < .001, *r* ^2^ = .60) (see Fig. 5). This result demonstrates that the effect is not limited to the peri-personal space and supports the hypothesis that one’s own body works as a spatial ruler in the conscious spatial perception of the entire visible world (see Fig. 6).

It could further be argued that if the own-body-size effect is reduced by distance, this reduction should be greater in the small-body condition because the apparent change in object distance (from 1.0 to 6.0 m) is larger when owning a small body. However, such an interaction between body size and distance was not found, *Z* = 0.457, *n* = 18, *p* = .65, which suggests that the own-body-size effect is genuinely similar for peri-personal space and far space.

In addition to these main findings, we noted that the apparent size of objects presented far from the cameras was significantly smaller than that of objects presented close to the camera for both body sizes, *Z*_small_ = 3.201, *n* = 18, *p* < .001, *r* ^2^ = .57; *Z*_large_ = 3.420, *n* = 18, *p* < .001, *r* ^2^ = .65. Because this effect was similar for both body,size conditions, it is most likely due to a systematic underestimation of distance when HMDs are worn (Swan, Jones, Kolstad, Livingston, & Smallman, 2007; Willemsen & Gooch, 2002). This underestimation is more profound for larger distances (Armbrüster, Wolter, Kuhlen, Spijkers, & Fimm, 2008; Williams, Rasor, & Narasimham, 2009) and might be caused by the restricted field of view (Wu, Ooi, & He, 2004). Furthermore, due to the size of the cameras, we were physically restricted to a lens-to-lens distance of 85 mm, but human interpupillary distance has an average of 63 mm (Dodgson, 2004). This large lens-to-lens distance likely caused an increase of binocular disparity, which resulted in an underestimation of distance. Importantly, this HMD-induced systematic underestimation of the sizes of objects presented far from the observer does not explain any of our main results, because our results were based on comparisons between conditions that were matched in this respect (comparing different body sizes).

### Combined analysis: comparing the own-body-size effect and visual size cues

In the present study, the effect of body size appeared to be entirely absent in the control condition with asynchronous visuotactile stimulation. This result is in contrast with the moderate effect of body size observed in the asynchronous control condition in our previous study when the body was visible during the object size test procedure (see Table 2 and van der Hoort et al., 2011). This discrepancy suggests that the artificial body can be used for direct visual comparison in the control condition if it is not occluded. Therefore, one might argue that under natural viewing conditions, the own-body-size effect and the body as a direct familiar-size cue work in concert in an additive fashion that involves two independent processes. Indeed, comparing the effect sizes of different experiments across our two studies suggests that using the artificial body as a direct familiar-size cue can also increase the effect of body size in the ownership condition, and this effect is added to the genuine own-body-size effect related to the feeling of having a certain body (*r* ^2^_Exp1a_ = .72, *r* ^2^_body visible_ = .91; see Table 2). Table 2 also suggests that controlling for fixation in Experiment 2 had very little, if any, influence on the own-body-size effect (*r* ^2^_Exp1a_ = .72, *r* ^2^_Exp2_ = .65).

**Table 2:** Effect sizes (*r* ^2^) of various conditions and experiments

	Methodological Design		*r* ^2^	
	Body Visibility During Object Presentation	Fixation Task	Body Ownership	Control
Experiment 6 (van der Hoort et al., 2011)	Visible	No	.**91**	.**72**
Experiment 1a	Occluded	No	.**72**	–
Experiment 1b*	Occluded	No	.82*	–
Experiment 2	Occluded	Yes	.**65**	–

* Effect size based on *Z*-score from nonparametric tests

To formally test these predictions, we conducted an analysis of the combined data from two experiments from the present study (Experiment 1a, no fixation, body occlusion; and Experiment 2, fixation, body occlusion) and one experiment from van der Hoort et al. (2011; Experiment 6, no fixation, no body occlusion). We used a general linear model with body ownership and body size as within-subjects factors and body visibility and fixation as between-subjects factors. First, we found a significant own-body-size effect [body size × body ownership: *F*(1, 46) = 61.341, *p* < .001]. Second, we found that body visibility has a significant effect on the effect of body size [body size × body visibility: *F*(1, 46) = 75.385, *p* < .001]. Interestingly, this effect of body visibility is independent of whether the participants experience ownership or not [body size × body ownership × body visibility: *F*(1, 46) = 0.207, *p* = .65]. Thus, it seems that the direct visual familiar-size cue of the body works independently of the own-body-size effect (defined by the body size × body ownership interaction) in affecting the perceived size of objects in the world. Furthermore, the influence of this familiar-size cue can be successfully eliminated by occluding the body, because there were no effects of body size in the control conditions of Experiment 1 or Experiment 2 (see Figs. 2 and 3). The same meta-analysis also confirmed that controlling fixation (Experiment 2) had no significant effect on the effect of body size [body size × fixation: *F*(1 ,46) = 0.341, *p* = .56] or on the own-body-size effect itself [body size × body ownership × fixation: *F*(1, 46) = 0.296, *p* = .59]. The effect of fixation on the effect of body visibility (body size × body visibility × fixation*)* could not be assessed with certainty because the analysis did not include a “fixation, no body occlusion” condition. However, this interaction is not relevant for the effect of body ownership and, thus, is beyond the scope of this study. Taken together, the results of the meta-analysis support our conclusion that the very experience of one’s own body influences visual spatial perception independently of the body as a visual familiar-size cue.

## Discussion

We used a full-body illusion that allowed us to manipulate the feeling of ownership of different-sized artificial bodies (see also van der Hoort et al., 2011) and subsequently tested object size perception. This method ensures that, for the same artificial body, the visual input is identical during body ownership conditions (i.e., synchronous touching) and control conditions (i.e., asynchronous touching). Therefore, the differences of object size perception between these conditions cannot be explained by traditional visual cues that are known to influence size and distance perception, such as object size on the retina, binocular disparity, texture gradient, linear perspective, and accommodation (see also van der Hoort et al., 2011), but, instead, can be attributed only to a difference in body ownership. In this study, we examined the relative contribution of two possible mechanisms by which body ownership of different-sized bodies could change object size perception: (1) Body ownership increases the use of the body as a familiar-size cue, and (2) body ownership causes a body-based rescaling of external space, after which the body itself is no longer needed to be seen directly for changes in object size perception to occur. To this end, we first used a novel methodological approach that eliminated any potential effect of using a body in view for direct visual comparison by occluding the body before the object test phase. Next, we added another methodological manipulation to eliminate the possible contribution of various fixation patterns by keeping fixation constant across the conditions. We found that the own-body size survived both of these manipulations and can, therefore, not be explained by familiar-size cues from the artificial body. Occluding the body before presenting the target objects decreased the effect of body size, but this seemed to be independent of body ownership. The additional control for fixation did not change the participants’ size estimations. The latter finding has multiple implications. First, by controlling for fixation, the extent to which the participants attend to the artificial body is similar across the various conditions. Because this did not change the results, attention as a confounder cannot have produced the own-body-size effect. In addition, this manipulation ensures that the influence of oculo-motor information on size perception is controlled for. Let us assume that during the period of visuotactile stimulation in the previous study (van der Hoort et al., 2011) and Experiment 1, the participants looked toward the touches applied to the legs of the artificial body. Upon object presentation, this would lead to eye *divergence* in the small-body condition and eye *convergence* in the large-body condition. These different eye movements can potentially affect the perception of depth (Brenner & Van Damme, 1998) and, hence, the perceived object size. When ownership of the artificial body is experienced, these eye movements might be more informative because they can be interpreted in terms of our body—the target object is further away than my legs versus closer by than my legs—instead of just shifting from one object (artificial body) to the next (target object). If this were true, the identical fixation during visuotactile stimulation and, thus, the eye-vergence in both the body size conditions in Experiment 2 would diminish the effect of ownership on object size perception. Because we did not observe such an effect, it is safe to assume that eye vergence plays no role in the own-body-size effect.

These results suggest that the own-body-size effect depends on a rescaling of external space that lasts for at least 5–6 s after the body is no longer visible. In addition, our results demonstrate that the own-body-size effect on visual object size perception is equally strong in space that is near the body and space that is far from the body, which suggests that the multisensory experience of owning a certain-sized body has a general effect on visuospatial perception. On the basis of our results, we propose a parsimonious model in which the central construct of external space depends on the multisensory representation of one’s own body.

It is always difficult to produce conclusive results in a single study; thus, we must consider the possible confounding factors and methodological limitations of the present study. We manipulated visuotactile synchrony to produce differences in the sense of body ownership between the experimental condition and the control condition while keeping visual information identical. This manipulation caused an effect on visual size perception. A critical reader may suggest that with the present set of experiments, we cannot distinguish between the contribution of low-level multisensory synchrony and the resulting sense of body ownership, since both phenomena consistently co-occurred. Multisensory synchrony in the absence of body ownership (e.g., replacing the artificial bodies with wooden blocks) would be needed to exclude the theoretical possibility that multisensory stimulation in isolation could cause the world to rescale. However, although low-level synchrony without body ownership could theoretically explain part of our findings, the fact that stronger subjective body ownership ratings were associated with stronger own-body-size effects shows that it cannot account for the own-body-size effect entirely.

It is also important to mention that because we solely manipulated visuotactile synchrony, the present study cannot assess the relative contribution of other necessary elements for the full-body illusion—that is, seeing a humanoid-shaped body and having a first-person perspective (Petkova & Ehrsson, 2008; Petkova et al., 2011). Furthermore, because the degree to which the full-body illusion is experienced in a view-only condition has never been tested for our setup, we did not include such a condition (but for findings from virtual reality research, see Maseli & Slater, 2013). We can therefore not determine the additional value of visuotactile synchrony or the disruptive effect of visuotactile asynchrony, as compared with seeing an artificial body from a first-person perspective without touch. But on the basis of the results, we suggest that any distortion of body ownership would diminish the own-body-size effect and any enhancement of body ownership would increase the own-body-size effect.

Another conceptual issue relates to the visibility of the testing room. When the test objects were presented, the participants could no longer see the artificial body, or any body for that matter, and so the body could not be used for direct visual comparison. However, the participants could see other familiar objects in the background of the testing room (the door, a table, and a chair). However, these familiar-size cues should work *against* the effect under investigation if they had any effect. The fact that we obtained a reproducible and robust own-body-size effect in such a natural environment strengthens the claim of a dominant role of the own body in scaling visuospatial perception.

A final methodological concern is whether we can fully exclude a possible contribution of fixation differences because we could not register eye movements with the present HMD system due to technical constraints. However, the same own-body-size effect was observed in two test situations that differed quite substantially in terms of their fixation instructions. Therefore, we believe it is reasonable to conclude that the differences of fixation cannot explain the present own-body-size effect.

Thus, the own-body-size effect in Experiment 2 can be attributed only to a rescaling of external space to body size that is caused by body ownership and in which new objects are rescaled accordingly without the need for a visible body.

The *own-body-size effect* reflects the interaction between body size and body ownership; that is, objects appear larger in the small-body condition and smaller in the large-body condition but only when the participants experience ownership of the artificial bodies. It is noteworthy to repeat that in the present study, the effect of body size on object size perception was entirely absent in the asynchronous control condition (i.e., when the participants did not experience ownership of the artificial body). This finding is in contrast with the small but significant body size effect in the asynchronous control condition we reported in our previous study, in which the artificial body was still present in the visual scene during the presentation of the target objects and the subsequent size estimations (see Table 2 and van der Hoort et al., 2011). This suggests that if the artificial body is not occluded in the control condition, it can be used as a direct familiar-size cue and, thereby, influence visual size perception independently of the own-body-size effect. Indeed, the formal meta-analysis (see the “Results” section) showed that the main effect of body visibility adds to the own-body-size effect but was independent of whether participants experienced ownership. Thus, in the present study, familiar-size cues have been completely eliminated by occluding the body, but the own-body-size effect remained intact.

The perceived sizes of the test objects in the small body (80 cm) is between 126 % and 220 % of the perceived sizes in the large body (400 cm), depending on the experiment listed in Table 2, instead of the 500 % that would theoretically be expected. This discrepancy shows that retinal information from objects still contributes to size perception and that the influencing effect from the own body is not entirely overriding this. Participants have lifelong experience with their real bodies and a similarly strong association between retinal object size and perceived object size, which could not be reset completely in the described experiments wherein participants switched between different body sizes roughly every minute.

An important conceptual issue relates to whether the size of one’s own body influences only visuospatial perception in the space immediately surrounding the body or whether the effect generalizes to the entire spatial layout, including space far from the observer. In line with our hypothesis, the present results show that the own body affects visual size perception for objects presented near the body and objects presented far from the body to a similar degree (*r* ^2^_Near_ = .70 and *r* ^2^_Far_ = .60; no significant effect of distance on own-body-size effect; see the “Results” section, Experiment 3). Thus, the own-body-size effect seems to be equally effective in peri-personal space and far extra-personal space. From this observation, we suggest that the own-body-size effect is not restricted to the role of vision in guiding limb action (Jeannerod, Arbib, Rizzolatti, & Sakata, 1995) or to the recalibration of visuotactile receptive fields in the peri-personal space (Brozzoli, Gentile, & Ehrsson, 2012; Maravita & Iriki, 2004; Spence et al., 2004) because neither of these conditions apply to far external space. Rather, the sense of the own body affects visuospatial perception in general. This conclusion is consistent with the illusion-induced changes in perceived distance to objects that we reported in our previous study (Experiments 9 and 10 in van der Hoort et al., 2011) and with the subjective experience of the illusion when participants are asked to freely describe how they experienced the test situation (Experiment 5 in van der Hoort et al., 2011).

Taken together, the present experiments show that the multisensory perception of a certain-sized body as one’s own (i.e., the feeling of body ownership) is a powerful, nonvisual cue that recalibrates object size perception. Through what mechanism does body ownership of different-sized bodies change the perceived size of target objects in the experiments we have described here? We theorize that first the entire visual layout is rescaled to the body. After that, the body no longer needs to be visible, because newly presented objects can now be scaled to this rescaled visual scene. In other words, changing the spatial representation of the body rescales the spatial representation of external space accordingly, which, in turn, causes changes in the perception of the sizes and distances of objects (Fig. 6). Importantly, a different-sized body that is not our own (i.e., in the control condition) can be used as a familiar-size cue but does not alter the spatial representation of the body, and therefore, no change in space perception occurs.

In our view, the multisensory representation of the own body changes the perception of external space because size and distance are meaningful only in relation to our own body size. For example, for somebody with a large body, an object at a certain location in space is seen as being five body sizes away and a quarter body size large, but for somebody with a small body, the same object is perceived as ten body sizes away and half a body size large. This hypothesis is in line with the ideas of several theoreticians who have claimed that there is something fundamental to owning a certain-sized body. For example, owning a different-sized body might change space perception because it changes the possible actions (i.e., affordances) of the environment (Gibson, 1986). In the small-body illusion, an object looks larger because the necessary movement or effort to interact with that object increases (Proffitt et al., 2003).

We can also look at our findings from a more mechanistic level. The change in visual perception of external space in the present study is caused by body ownership illusions. Body ownership illusions are induced by the synchronous visual and tactile stimulation of one’s real body and a visible artificial body (Botvinick & Cohen, 1998; Ehrsson, Holmes, & Passingham, 2005; Ehrsson, Spence, & Passingham, 2004; Petkova & Ehrsson, 2008; Petkova et al., 2011; Tsakiris & Haggard, 2005). Initially, there is a spatial conflict between the visual, tactile, and proprioceptive signals, but this conflict is eliminated by recalibration of the various signals to achieve alignment (Ehrsson, 2012) due to the inherent tendency to eliminate multisensory conflicts and to bind information across the senses into meaningful percepts (Ernst & Banks, 2002; Stein & Stanford, 2008). The end result is a perceptual fusion of visual, tactile, and proprioceptive signals into a unified perception of a single owned body that constitutes the physical self (Ehrsson, 2012). This process involves the recalibration of all body-centered representations of space (Ehrsson, 2012), including visuotactile representations of peri-personal space (Brozzoli et al., 2012). Thus, in the present experiments, touch is felt at a specific location on the legs, and this tactile impression is matched with an observed touch at a specific location in space, which gives rise to the feeling that the felt touch and the observed touch are manifestations of the same single visuotactile object. Similarly, the visual impression of the two artificial legs and torso is matched to the participant’s proprioceptive sensation from these body parts. Thus, the participants experience a vivid perceptual illusion that the stimulated small or large body is their own.

In the next step, this change in the central multisensory body representation is translated into a change in the central representations of the entire spatial layout, which influences the perception of the sizes of objects far from the body. We argue that representation of external space in the brain is formed by a complex set of computations that are based on all available sensory information from the various sense modalities (Azañón, Longo, Soto-Faraco, & Haggard, 2010). The transformation from body-centered spatial frames of reference to allocentric (or world-centered) spatial reference frames is thought to represent a crucial stepping stone in the processes of generating this central construct of the external world (Burgess, 2006; Whitlock, Sutherland, Witter, Moser, & Moser, 2008). Therefore, we argue that the recalibration of body-centered representations of space onto small or large artificial bodies automatically produces an up-scaling or down-scaling of the external environment because egocentric and allocentric representations depend on the size of the observer. We illustrate this parsimonious idea in Fig. 6, which shows how changing the size of the multisensory body representation causes changes in the central representation of external space.

What is the neuronal basis of these interactions? The multisensory representation of the body (Ehrsson et al., 2004; Gentile, Petkova, & Ehrsson, 2011; Lloyd, Shore, Spence, & Calvert, 2002) in the human brain is implemented in multisensory areas in the premotor cortex and the intraparietal sulcus. Single neurons that have the capacity for multisensory integration have been found in the brains of nonhuman primates (Avillac, Ben Hamed, & Duhamel, 2007; Graziano, 1999; Graziano, Cooke, & Taylor, 2000; Graziano et al., 1997). Importantly, human fMRI experiments have shown that the BOLD signals in these areas closely follow the perceptual experience of the full-body illusion (Petkova et al., 2011). For visual information (and the size cues contained therein) to be interpreted relative to one’s own body, we propose the existence of a feedback loop from the intraparietal sulcus back to the parietal and occipital areas that are related to visuospatial object perception (Konen & Kastner, 2008; Kourtzi & Connor, 2011). In parallel, the body-centered multisensory information in the premotor and posterior parietal areas may affect all egocentric or allocentric representations of far space in the medial and lateral temporal cortex and the medial parietal cortex (Burgess, Maguire, & O’Keefe, 2002; Galati, Pelle, Berthoz, & Committeri, 2010; Kravitz, Saleem, Baker, & Mishkin, 2011; Maguire et al., 1998; Moser, Kropff, & Moser, 2008). In turn, recalibration of these spatial representations would affect the representation of the sizes of objects that are presented far from the body, and this would presumably occur via a feedback projection back to the parietal and occipital areas that compute object size.

## Conclusions

The present study has three main findings regarding the own-body-size effect—that is, the observation that objects appear larger to smaller observers and smaller to larger observers. First, the multisensory experience of owning a certain-sized body is an independent contributor to the own-body-size effect from the mere usage of the (own) body in direct visual comparison. Second, differences in fixation make no contribution to the own-body-size effect. Third, the own-body-size effect applies to both the peri-personal space and far external space. Moreover, the own-body-size effect affects both verbal and behavioral measures of size perception, which is in line with previous findings (van der Hoort et al., 2011). Our findings suggest an important role of the size of the body we own in the perception of the sizes of objects that is independent of traditional visual cues and independent of an object’s distance from the observer. This role of our body in visual space perception is an important addition to the traditional visual cue approach and aids understanding of the fundamental embodied nature of visual perception.
